# Exploring Patterns of Palmar Hyperlinearity in Pediatric Population With Atopic Dermatitis or Ichthyosis Vulgaris Attending a Tertiary Care Hospital in Jaipur, India

**DOI:** 10.7759/cureus.103192

**Published:** 2026-02-08

**Authors:** Aditi Goyal, Reshu Gupta, Riya Singhani, Prahlad Dhakar, Siddhi Bhardwaj, Ayushmaan Parmar

**Affiliations:** 1 Department of Dermatology, RUHS College of Medical Sciences, Jaipur, IND; 2 Department of Physiology, RUHS College of Medical Sciences, Jaipur, IND; 3 Department of Medicine, RUHS College of Medical Sciences, Jaipur, IND

**Keywords:** atopic dermatitis, filaggrin, hyperlinear palms, ichthyosis vulgaris, pediatric dermatology

## Abstract

Introduction: Ichthyosis vulgaris (IV) and atopic dermatitis (AD) are skin diseases occurring in children and having important quality-of-life implications. Palmar hyperlinearity has been characterized as a visible sign of skin barrier abnormality in these disorders.

Methods: In this hospital-based cross-sectional study, children aged six months to 18 years with a confirmed diagnosis of AD, IV, or both were assessed for five pre-defined palmar patterns. Patterns were graded as hyperlinear or non-hyperlinear, and their correlation with disease groups was examined. Inter-rater reliability was evaluated in a subset.

Results: Out of the 206 recruited participants, 121 children (58.7%) had hyperlinear patterns. Frequent cross-hatch was the most common hyperlinear pattern overall, found in relation to isolated AD predominantly. Thick perpendicular lines were most prevalent in IV-only cases, and frequent diamond patterns occurred in children with both AD and IV. Classification was found to have considerable inter-observer agreement (Fleiss Kappa statistic= 0.62).

Conclusions: Patterns of palmar hyperlinearity in AD and IV are different. Detection can be a low-cost adjunct for the early diagnosis and phenotyping, particularly in cases where genetic analysis is not accessible.

## Introduction

Atopic dermatitis (AD), a skin disease, is diagnosed based on the Hanifin-Rajka criteria. It is chronic, relapsing, pruritic, and inflammatory [1,2], with filaggrin (FLG) loss-of-function (LoF) mutations as the main genetic risk factor (with ~50% of AD patients carrying these alleles) [3,4], although environmental factors also play a role [3,5-7]. The mutations are associated with early-onset, severe, and persistent phenotypes of AD [6]. The prevalence of pediatric AD varies from 3.1-7.21% in India [4]. Most cases have a childhood onset of AD, often coexisting with asthma, allergic rhinitis, or vitiligo [2,3,6-8].

Ichthyosis vulgaris (IV), a common inherited ichthyosis (>95% of cases), typically presents in infancy. It is characterized by xerosis, keratosis pilaris, and palmar hyperlinearity [3,9-11]. IV affects ~one in 250 individuals and also shows a marked filaggrin (FLG) deficiency [12,13]. Even though AD and IV are clinically distinct, they share an overlapping pathophysiology due to compromised epidermal barriers, with FLG LoF mutations strongly implicated in both disorders [5,14,15]. Routine genetic testing is not feasible in low-resource settings, making phenotypic markers important as practical diagnostic tools.

Palmar hyperlinearity (HLP) is a minor diagnostic criterion for AD and a visible clinical indicator of FLG mutations. It is defined as ≥five prominent lines >1 cm across the thenar eminence [16,17]. Five palmar patterns-prominent diamond, thick perpendicular, extensive cross-hash, fine cross-hash, and fine perpendicular-have been identified in Bangladeshi populations by a previous study [18]. Patterns might potentially relate to disease severity or subtype, but inconsistent terminology has limited their clinical utility. Standardized classification based on line depth and pattern complexity was done in this study. Fine cross-hash and fine perpendicular were grouped as non-hyperlinear palms (non-HLP), while prominent diamond, thick perpendicular, and extensive cross-hash were grouped as hyperlinear palms (HLP).

The objectives of this study are to identify various HLP patterns in the Indian pediatric population with AD and/or IV; postulate correlation, if any, of the patterns with the underlying disease, and identify any potential disease-specific HLP patterns. This might offer a low-cost method for early detection and risk stratification, especially valuable in primary care and dermatology clinics where genetic testing is unavailable.

## Materials and methods

Ethical clearance was provided by the Institutional Ethical Committee of RUHS College of Medical Sciences, Jaipur. Patients were recruited voluntarily, with confidentiality strictly maintained, and no invasive procedures were performed. 

Children (aged six months to 18 years) who were diagnosed with AD or IV were included, and the diagnosis was confirmed using standard criteria independently by three dermatologists. Children with conditions such as seborrheic dermatitis, psoriasis, scabies, tinea, acrodermatitis enteropathica, or who engaged in manual labor were excluded to prevent misclassification.

A sample size of 206 participants was calculated using a two-proportion comparison formula, requiring at least 95% confidence (Z=1.96), 80% power (Z=0.84), and a 12% group difference.

**Figure 1:**
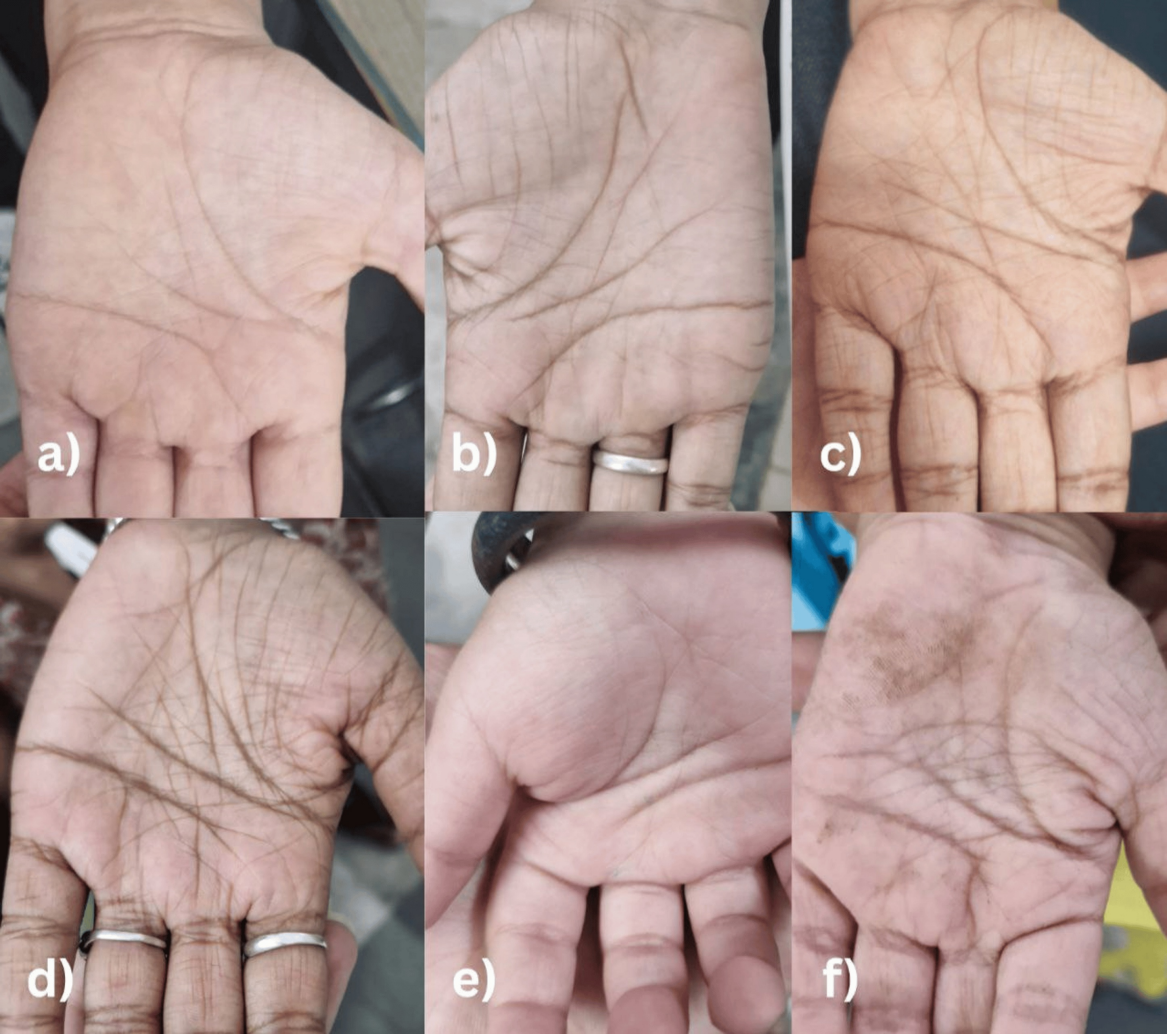
High-resolution clinical photographs demonstrating the five categorized palmar patterns. Non-hyperlinear patterns: (a) Fine cross-hatch (e) Fine perpendicular hyperlinear patterns: (b) Prominent diamond/vertical (c) Extensive cross-hatch (d) Prominent diamond (f) Thick perpendicular

Written informed consent was obtained from parents or legal guardians for clinical examination and for capturing photographic images of the participants’ palms using iPhone 14’s rear dual-camera system (12 MP f/1.5 wide) with sensor-shift OIS for high-clarity imaging for documentation and publication purposes. Palms were examined and classified as HPL or non-HPL, which were further classified as: non-HPL- fine perpendicular, fine cross-hash; HPL- extensive cross-hash, thick perpendicular, and prominent diamond (vertical) (Figure 1) [18].

A pre-designed, pilot-tested proforma (Appendix Table 3) was used to collect data, including demographics (age, sex, BMI, family history, immunisation, and clinical diagnosis). All 206 participants were assessed by a single trained dermatologist to ensure consistency and to evaluate inter-rater reliability; a subset of 50 cases was reassessed by two independent, blinded dermatologists individually. In case of any disagreement, the opinion of the initial dermatologist was considered final.

Data were analysed using descriptive and inferential statistics. Palm pattern correlation with AD or IV was assessed using Pearson’s chi-square test. Inter-rater reliability was measured using Fleiss’ kappa, with significance set at p < 0.05. Microsoft Excel and IBM SPSS Statistics for Windows, Version 28.0 (IBM Corp., Armonk, NY, USA) were used for statistical analysis.

## Results

A total of 206 children with a mean age of 10.2 ± 4.3 years were enrolled: 107 males (51.9%) and 99 females (48.1%). Diagnoses showed 143 (69.4%) with AD, 22 (10.7%) with IV, and 41 (19.9%) with both AD+IV (Table 1).

**Table 1:** Baseline demographic and clinical characteristics of children diagnosed with AD or/and IV, stratified by HPL pattern Values represent mean (SD) for continuous variables and number (%) for categorical variables. Age and BMI were analyzed using one-way ANOVA (F statistic reported). Sex, family history, and diagnosis groups were compared using Pearson’s chi-square test (χ² statistic reported). AD: Atopic Dermatitis; IV: Ichthyosis Vulgaris; HOF: History of Illness in Family

Characteristics	Fine Cross Hatch	Fine Perpendicular	Extensive Cross Hatch	Thick Perpendicular	Prominent Diamond	P-values	Test Statistics
	( n = 52 )	( n = 33 )	( n= 51 )	( n = 30 )	( n = 40 )		
Age	9.23 (5.0)	7.90 (5.2)	8.30(5.6)	8.09(4.6)	8.10(4.4)	0.726	F = 0.513 df = (4, 201)
BMI	20.48 (1.7)	20.79 (2.1)	20.41 (2.2)	20.37 (2.2)	20.75(2.5)	0.869	F = 0.313 df = (4, 201)
Male	25 (48.1%)	20 (60.6%)	27 (52.9%)	11 (36.7%)	24 (60.0%)	0.27	χ²=5.16
Female	27 (51.9%)	13 (39.4%)	24 (47.1%)	19 (63.3%)	16 (40.0%)		
HOF Illness – Yes	15 (28.8%)	8 (24.2%)	21 (41.2%)	7 (23.3%)	14 (35.0%)	0.36	χ²=4.33
HOF Illness – No	37 (71.2%)	25 (75.8%)	30 (58.8%)	23 (76.7%)	26 (65.0%)		
AD	46 (88.5%)	30 (90.9%)	37 (72.5%)	17 (56.7%)	13 (32.5%)	<0.001	χ²=70.30
IV	4 (7.7%)	2 (6.1%)	4 (7.8%)	9 (30.0%)	3 (7.5%)		
AD and IV	2 (3.8%)	1 (3.0%)	10 (19.6%)	4 (13.3%)	24 (60.0%)		

HLP patterns were classified into five categories: Extensive cross-hash, thick perpendicular, and prominent diamond were HLP, while fine perpendicular and fine cross-hash were non-HLP (Figure 1).

**Figure 2:**
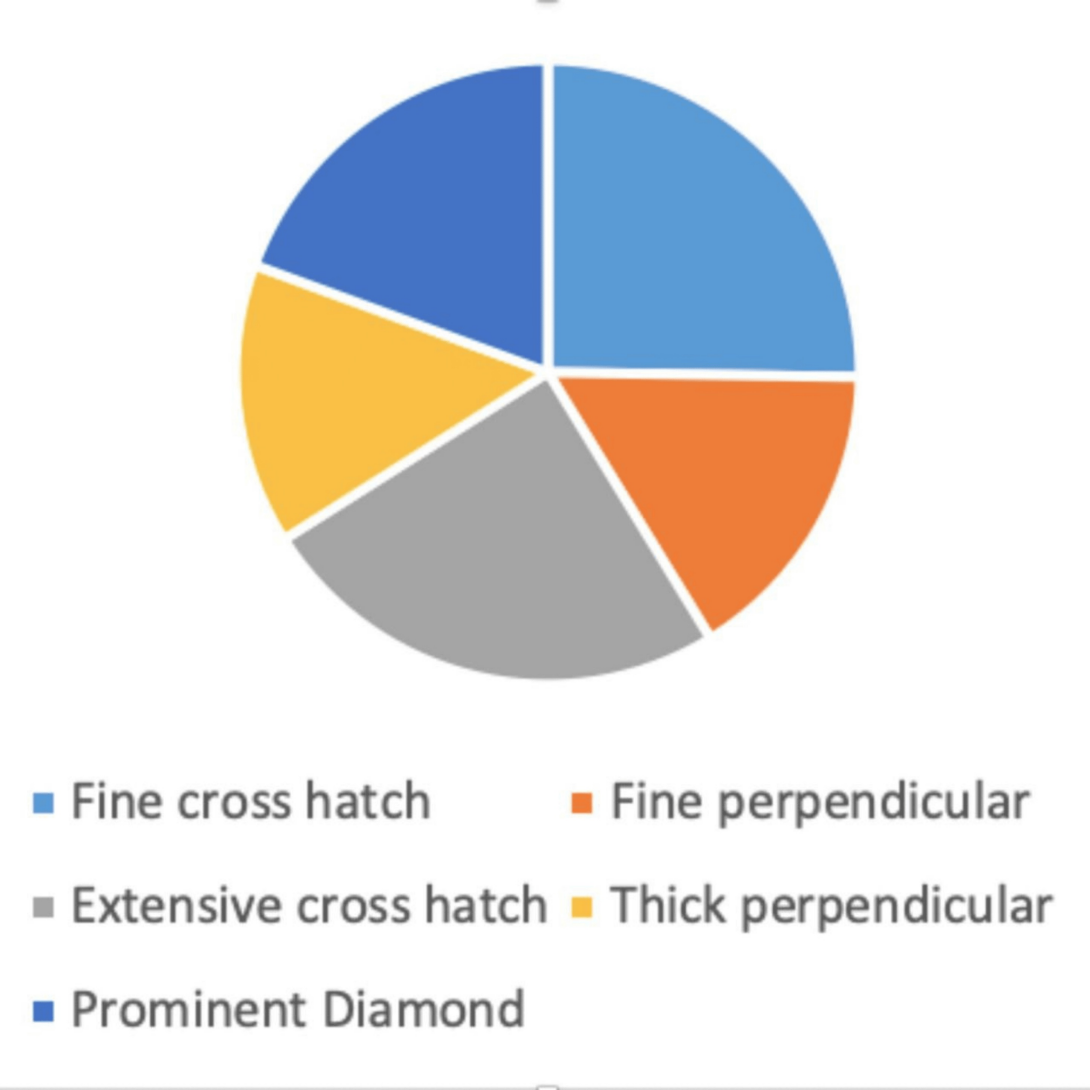
Distribution (%) of palmar patterns among children aged 6 months to 18 years attending the Departments of Dermatology and Pediatrics (n = 206).

The pie chart illustrates the proportional representation of each palm pattern category: Fine cross-hatch, fine perpendicular, extensive cross-hatch, thick perpendicular, and prominent diamond, based on the total study sample (Figure 2).

Extensive cross-hash: Most common HLP overall, found in 51 children. Of these, 37 (72.5%) had AD alone, four (7.8%) had IV alone, and 10 (19.6%) had AD+IV. This was the most frequent pattern in isolated AD (Table II).

Thick perpendicular: Found in 30 children, with 17 (56.7%) having AD and nine (30%) having IV alone. This was the dominant pattern in isolated IV.

Prominent diamond: Observed in 40 children. Most (60%) had both AD and IV, 32.5% had AD alone, and 7.5% had IV alone, indicating a compounded barrier defect.

Among non-HLP, fine cross-hash was most common (52 cases), 88.5% of which were in AD. Fine perpendicular was seen in 33 children, with 30 cases of AD (Table 2).

**Table 2:** Prevalence (%) of hyperlinear and non-hyperlinear patterns in diagnostically confirmed groups of AD, IV, AD and IV ( N=206). Values are shown as a number (%). Percentages correspond to the proportion of each pattern observed within each disease category. AD: Atopic Dermatitis; IV: Ichthyosis Vulgaris.

Patterns	Disease		
	AD	IV	AD+IV
Non Hyperlinear Patterns	( n = 143 )	( n = 22 )	( n = 41 )
Fine Cross -hash	46 (88.5%)	4 (7.7%)	2 (3.8%)
Fine Perpendicular	30 (90.9%)	2 (6.1%)	1 (3.0%)
Hyperlinear Patterns			
Extensive Cross -hash	37 ( 72.5%)	4 (7.8%)	10 (19.6%)
Thick Perpendicular	17 (56.7%)	9 (30.0%)	4 (13.3%)
Prominent Diamond	13 (32.5%)	3 (7.5%)	24 (60.0%)

The correlation between palm pattern and diagnosis was statistically significant (p= 0.012). No significant association was found with age, sex, or BMI. Inter-rater reliability was substantial (Fleiss κ = 0.62).

There were classification difficulties in distinguishing thick perpendicular lines from prominent diamond patterns. The features more closely resembled the vertical hyperlinearity described by Brown (2009), characterised by overlapping lattice-like lines. Notably, this vertical pattern was not described in the 2023 classification by Thomas [18,19].

No significant correlations were found between palm patterns and age (F=0.51; df=(4, 201); p=0.72), BMI (F=0.313; df=(4, 201); p=0.86), sex (p=0.27; χ²=5.16), or family history (p=0.36; χ²=4.33). However, a strong association existed between disease type and palm pattern distribution (p<0.001; χ²=70.30).

## Discussion

This study highlights distinct associations between specific HLP patterns and underlying dermatological diagnoses in Indian children.

Extensive cross-hash was most strongly linked to isolated AD, with over 70% of children showing this pattern. It likely reflects Th2/Th22 driven chronic inflammation and barrier disruption characteristic of moderate-to-severe AD, aligning with earlier reports connecting complex palmar patterns to active disease and filaggrin deficiency [18,19].

Thick perpendicular showed the strongest association with IV alone, suggesting this pattern may reflect structural corneocyte variation and diminished desquamation inherent to IV rather than acquired inflammatory damage. Prominent diamond was most frequent in children with both AD and IV, indicating a compounded genetic and inflammatory barrier defect. Its rarity in isolated conditions suggests it may represent later-stage or more severe disease phenotypes. The interpretations are hypothesis generating and consistent with documented biological findings, though only genetic and biophysical studies can establish causality.

Non-HLP patterns (fine cross-hash and fine perpendicular) were primarily found in mild or early AD, indicating minimal barrier involvement.

This is the first Indian study demonstrating reproducible, disease-specific correlations of HLP patterns with pediatric dermatoses. Inter-rater agreement was substantial (Fleiss κ = 0.62), confirming the reliability of visual pattern recognition in clinical settings. However, overlap between vertical and diamond patterns, as noted by Thomas et al. [18], suggests a need for standardized image databases or AI-assisted classification for improved diagnostic precision.

The findings of our study partially align with those of Thomas et al(2023), particularly in demonstrating that certain palmar patterns tend to associate with specific underlying skin pathologies. However, the vertical pattern, which was distinctly identifiable in our Indian Pediatric population, was not described as a standalone configuration in their South-Asian cohort [18]. Furthermore, another landmark study on palmar patterns reported a significantly higher prevalence of prominent hyperlinear patterns in patients with compound barrier defects. The observation was consistent with our results, as the most complex “Prominent Diamond” pattern was predominantly seen in children affected with AD+IV [19].

Key strengths of this study include its standardized pattern classification and demonstrated inter-rater reliability, supporting the reproducibility of palmar pattern assessment in routine clinical practice.

Limitations include the absence of genetic testing to directly confirm FLG mutation associations and the single-center design, limiting generalizability among various geographic and ethnic populations. Future studies integrating genetic testing and larger multicentric cohorts are needed to validate these findings. Palmar line depth and structural analysis should also be further explored, especially in IV.

Routine palm examination could be integrated into pediatric dermatology evaluations, improving early detection, personalized care, and quality of life, particularly where genetic testing is inaccessible.

## Conclusions

This research illustrates that certain patterns of palmar hyperlinearity are efficacious, low-budget clinical indicators for discriminating AD, IV, and overlapping disease in children. Extensive cross-hatch, thick perpendicular and prominent diamond are strongly correlated with AD, IV and AD + IV, respectively, indicating that palmar line morphology may reflect disease-specific skin barrier changes. Inter-rater agreement was high (Fleiss k=0.62), supporting the reproducibility of this palm-based classification. Standardised assessment of pattern inclusion in routine dermatologic assessment might enhance early detection, disease stratification, and patient care in paediatric dermatology clinics.

## Appendices

**Table 3:** Proforma: Exploring patterns of Palmar hyperlinearity in pediatric population with atopic dermatitis or ichthyosis vulgaris.

Section	Field	Details / Options
Participant Details	Date	
	Participant ID	
	Name of Participant	
	Name of Guardian	
	Age (years)	
	Gender	☐ Male ☐ Female ☐ Other
	Address	
Anthropometry	Weight (kg)	
	Height (cm)	
	BMI	
Family & Medical History	Family history of Atopic Dermatitis	☐ Yes ☐ No
	If Yes, Relation	
	Family history of Ichthyosis vulgaris	☐ Yes ☐ No
	If Yes, Relation	
	Family history of other illnesses	☐ Yes ☐ No
	If Yes, Specify	
	Associated diseases with AD	☐ Urticaria ☐ Allergic rhinitis ☐ Asthma ☐ Food allergy ☐ Other: __________
Diagnosis	Primary diagnosed skin disease	☐ Atopic Dermatitis ☐ Ichthyosis vulgaris ☐ Both
	Age at diagnosis of AD	
	Age at diagnosis of IV	
	Other skin diseases	☐ Yes ☐ No
	If Yes, Mention	
Palm Examination	Dominant Pattern Type (Thomas BR et al.)	
	Fine perpendicular	☐ Examiner 1 ☐ Examiner 2
	Fine cross-hash	☐ Examiner 1 ☐ Examiner 2
	Extensive cross-hash	☐ Examiner 1 ☐ Examiner 2
	Thick perpendicular	☐ Examiner 1 ☐ Examiner 2
	Prominent diamond	☐ Examiner 1 ☐ Examiner 2
	Hyperlinearity Right Palm	☐ Yes ☐ No
	Hyperlinearity Left Palm	☐ Yes ☐ No
Examiner Details	Examiner 1 (Primary): Name	
	Designation	
	Signature	
	Date	
	Examiner 2 (Independent assessor): Name	
	Designation	
	Signature	
	Date	
